# Contrast extravasation on Dyna-CT as a predictor of malignant brain edema after mechanical thrombectomy for acute anterior circulation large vessel occlusion

**DOI:** 10.3389/fneur.2026.1784893

**Published:** 2026-03-06

**Authors:** Qingjie Chi, Wenlu Shi, Jinhua Qian, Wenbin Ding, Tianhao Zhang, Dongjian Chen, Tianle Wang, Zhuo Chen, Li Zhu

**Affiliations:** 1 Department of Intervention, Nantong First People's Hospital, Southeast University, Nantong, Jiangsu, China; 2 Department of Radiology, Affiliated Nantong Clinical College of Nantong University, Nantong First People's Hospital, Nantong, Jiangsu, China; 3 Department of Neurology, Nantong First People's Hospital, Nantong, Jiangsu, China; 4 Department of Radiology, Nantong First People's Hospital, Southeast University, Nantong, Jiangsu, China

**Keywords:** contrast extravasation, Dyna-CT, malignant brain edema (MBE), mechanical thrombectomy (MT), stroke

## Abstract

**Background:**

Contrast agent extravasation detected on immediate post-thrombectomy flat-panel detector computed tomography (Dyna-CT) may reflect blood–brain barrier disruption and microcirculatory dysfunction following mechanical thrombectomy (MT) for acute anterior circulation large vessel occlusion (AC-LVO). This study aimed to determine whether contrast extravasation on Dyna-CT after successful MT is associated with the subsequent development of malignant brain edema (MBE).

**Methods:**

A retrospective study was conducted in AC-LVO patients who underwent MT with successful recanalization between January 2020 and December 2023. Dyna-CT was performed immediately after MT, followed by serial head CT scans. MBE was defined as acute cerebral swelling with a midline shift ≥5 mm accompanied by radiological signs of brain herniation. Patients were divided into MBE and non-MBE groups. Multivariable logistic regression analyses were performed to identify independent predictors of MBE and construct a predictive model.

**Results:**

A total of 174 patients (median age, 71 years) were included, with an MBE incidence of 23.6% (41/174). Core infarct volume, baseline NIHSS score, collateral circulation, number of thrombectomy passes, and contrast extravasation were independent predictors of MBE (all *P* < 0.05). The combined predictive model showed excellent discrimination (AUC = 0.90, 95% CI: 0.85–0.96).

**Conclusion:**

Immediate Dyna-CT detection of contrast extravasation serves as a valuable imaging biomarker for predicting MBE in AC-LVO patients after successful MT, offering timely guidance for early risk identification and individualized management.

## Introduction

Mechanical Thrombectomy (MT) has become a cornerstone therapy for acute anterior circulation large vessel occlusion (AC-LVO) stroke (1–5). By restoring cerebral perfusion and salvaging the ischemic penumbra, MT could lead to an improvement in neurological outcomes. However, despite continuous advancements in thrombectomy techniques and an approximately 85% rate of successful revascularization, nearly 40% of patients still experience unfavorable functional outcomes at 90 days (mRS ≥ 3) after the procedure (6). Among the determinants of poor prognosis, the development of malignant brain edema (MBE) stands out as a devastating complication associated with high mortality and severe neurological deterioration (7, 8). The pathophysiology of MBE is multifactorial, involving a complex interplay of severe ischemia and hypoxia, disruption of the blood–brain barrier, microcirculatory dysfunction, accumulation of inflammatory mediators (9). The occurrence of MBE following MT is closely associated with infarct core size, a larger infarct core typically reflects more extensive irreversible ischemic injury and more severe disruption of cellular energy metabolism (10). In addition, reperfusion injury and contrast extravasation may further exacerbate endothelial damage and inflammatory cascades. Collectively, these processes precipitate rapid cytotoxic and vasogenic edema after MT, resulting in marked hemispheric swelling and midline shift within hours. Once established, it often responds poorly to conventional medical therapy—including osmotic agents and intracranial pressure–lowering measures—necessitating early recognition and proactive management. Timely surgical intervention, such as decompressive hemicraniectomy, may be life-saving in selected patients (11).

Non-contrast enhanced CT is currently the most widely available imaging modality for determining the presence of cerebral edema, and several NCCT-derived imaging biomarkers, including midline shift, cerebrospinal fluid displacement and net water uptake, have been widely applied to assess the risk of MBE (8, 12). However, the acquisition of these parameters often relies on postoperative or follow-up imaging, which require patient transfer to the radiology department and typically take approximately 6 h to complete, resulting in a considerable time delay. This temporal lag limits their utility for early risk stratification before overt clinical deterioration occurs and reduces their ability to guide timely intervention and treatment. In recent years, contrast extravasation following MT has emerged as a novel imaging biomarker (13, 14), showing promising potential in predicting postprocedural complications. This phenomenon is closely related to pathological processes such as blood–brain barrier disruption (15, 16), reperfusion injury (17), and venous outflow dysfunction (18), all of which are key mechanisms underlying the development of MBE

Notably, contrast extravasation can be visually identified on immediate postprocedural flat-panel detector computed tomography (Dyna-CT) scans as areas of high density within the infarcted region (19, 20). In comparison, dual energy computed tomography (DECT) enables more precise differentiation between contrast staining and hemorrhage through spectral separation techniques, but its examination is typically performed at a delayed stage. Logistical factors such as equipment availability, scanning schedule, and the potential risks of patient transfer limit its practicality for early postoperative evaluation. In contrast, Dyna-CT, integrated within the angiography suite, enables seamless, real-time imaging immediately after MT without the need for patient relocation. This modality offers bedside accessibility, operational efficiency, and immediate visualization of contrast extravasation, facilitating the early assessment of blood–brain barrier (BBB) integrity and microcirculatory impairment.

Although previous studies have demonstrated a strong association between contrast extravasation and postoperative MBE, systematic evidence on the clinical utility of immediate Dyna-CT in patients with AC-LVO following MT remains limited. Given that malignant brain edema may develop both as a consequence of extensive infarction and as a reperfusion-related phenomenon, this study retrospectively analyzed the clinical and Dyna-CT imaging data of AC-LVO patients who underwent MT to investigate the relationship between contrast extravasation and post-reperfusion MBE and to develop a predictive model for early identification of high-risk patients.

## Methods

### Study population

This retrospective cohort study consecutively enrolled patients who presented to the interventional department and stroke center of our hospital within 24 h of symptom onset between January 2020 and December 2024. All included patients had AC-LVO confirmed by computed tomography angiography (CTA), underwent perfusion assessment with computed tomography perfusion (CTP), and achieved successful recanalization following MT. Patients who voluntarily discharged themselves within 12 h after the procedure (*n* = 18) were excluded from the analysis. Immediately after the MT, all patients underwent Dyna-CT imaging, followed by DECT monitoring and subsequent follow-up scans as clinically indicated. In total, 174 patients met the eligibility criteria and were included in the final analysis. All diagnostic and therapeutic procedures were conducted in accordance with institutional protocols, with written informed consent obtained from each patient or their legally authorized representative.

Inclusion criteria were as follows: (1) age ≥ 18 years; (2) diagnosis of AC-LVO stroke, meeting the diagnostic criteria for acute ischemic stroke as defined in the Chinese Guidelines for the Diagnosis and Treatment of Acute Ischemic Stroke (2020); (3) time from symptom onset to intervention ≤24 h, with a pre-stroke modified Rankin Scale (mRS) score of 0–1, a National Institutes of Health Stroke Scale (NIHSS) score ≥ 6, and an Alberta Stroke Program Early CT Score (ASPECTS) ≥ 6. A small proportion of patients with ASPECTS 3–5 were included following individualized multidisciplinary evaluation when perfusion imaging demonstrated a limited infarct core volume (<50 ml); (4) completion of CTP imaging prior to MT, with successful reperfusion confirmed by a modified Thrombolysis in Cerebral Infarction (mTICI) grade of 2b or 3; (5) immediate postoperative Dyna-CT imaging performed under digital subtraction angiography (DSA), followed by the first DECT scan within 6–12 h after treatment; and (6) assessment of clinical outcome at 90 days using the mRS, with a score of 0–2 indicating a favorable neurological prognosis.

Exclusion criteria included: (1) pre-existing hemispheric structural abnormalities (e.g., hemorrhage, tumor) or radiologically confirmed hemorrhagic transformation after MT; (2) posterior circulation ischemic stroke; (3) severe hepatic or renal dysfunction; (4) known allergy to iodinated contrast agents; and (5) incomplete clinical or imaging data.

### Clinical information collection and assessment

Upon admission, baseline clinical characteristics were recorded, including age, sex, and medical history (e.g., hypertension, diabetes mellitus, dyslipidemia, coronary artery disease, and prior stroke). Additional variables collected comprised preoperative neurological status assessed by the NIHSS, preoperative imaging parameters—infarct core volume (ICV), ischemic penumbra volume (IPV), and collateral circulation—administration of intravenous thrombolysis prior to MT, procedural details (number of thrombectomy attempts), postoperative imaging findings of contrast extravasation, and functional outcomes evaluated by the mRS score at 90 days after the procedure.

The diagnosis of MBE (21) was established according to the following criteria: (1) Neurological deterioration or decline in consciousness, defined as an increase of ≥2 points in the total NIHSS score or ≥1 point in the consciousness subscore; and (2) Non-contrast head CT performed within 72 h after MT showing hypodense lesions involving >50% of the middle cerebral artery territory, accompanied by marked parenchymal swelling; and (3) Midline shift of the septum pellucidum or pineal gland ≥5 mm, with associated ventricular compression, effacement of cerebral sulci and gyri, and obliteration of the interpeduncular cistern.

### Surgical procedure and perioperative management

**Figure 1:**
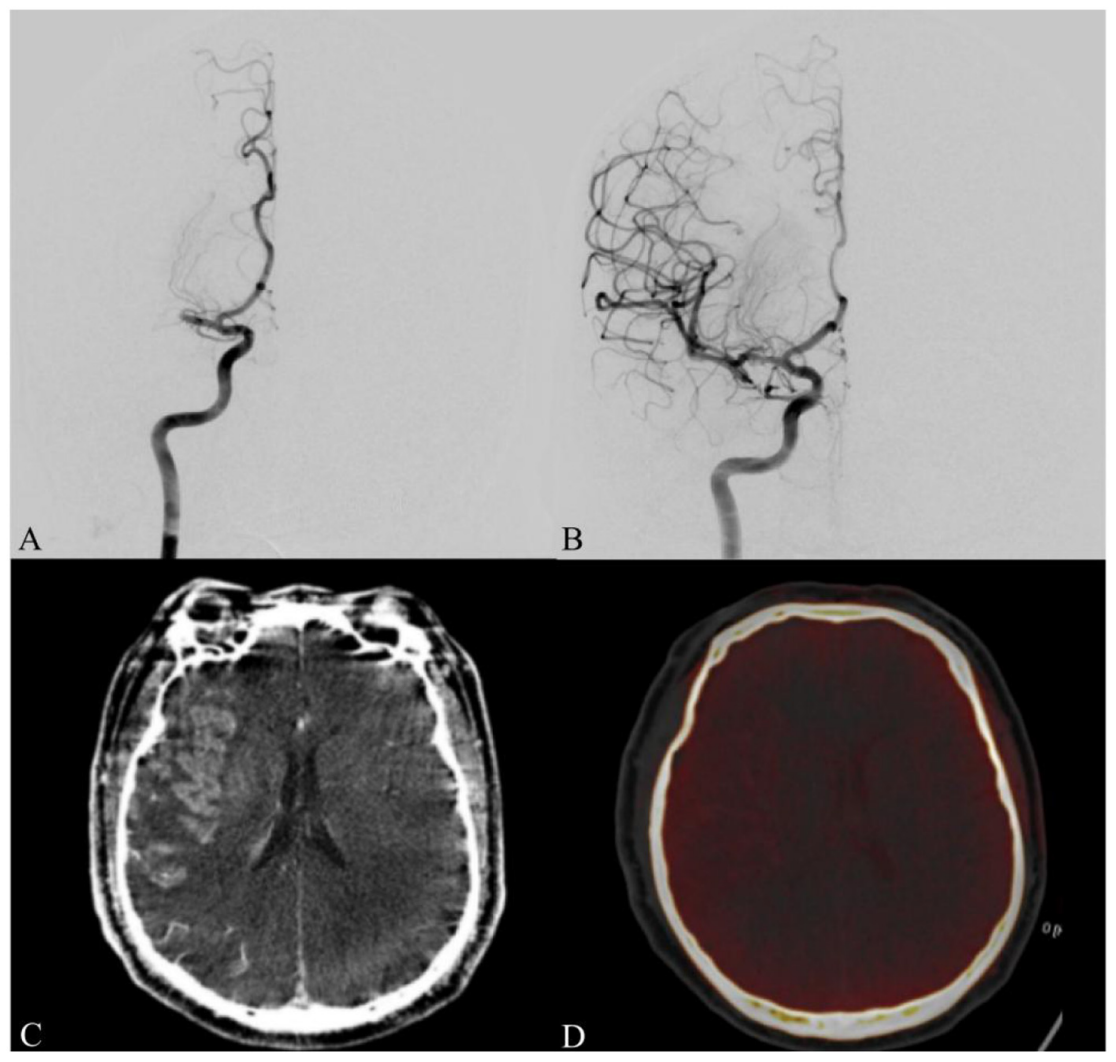
A patient with occlusion of the right middle cerebral artery M1 segment. **(A)** Preoperative digital subtraction angiography (DSA) shows no antegrade flow (mTICI 0). **(B)** Postoperative angiography demonstrates complete reperfusion (mTICI 3) following mechanical thrombectomy. **(C)** Immediate post-thrombectomy Dyna-CT images showing contrast agent extravasation and retention in the brain parenchyma. **(D)** Post-thrombectomy dual-energy CT confirmed that the ipsilateral hyperdensity represented contrast extravasation, with mild cerebral edema and slight leftward midline shift. DSA, digital subtraction angiography; mTICI, modified thrombolysis in cerebral infarction.

All mechanical thrombectomy (MT) procedures were performed using a Siemens Artis zee digital subtraction angiography (DSA) system (Siemens Healthineers, Germany). Interventions were conducted by experienced neurointerventionalists with over 15 years of clinical practice. Anesthesia type—local or general—was selected based on patient cooperation, clinical condition, and procedural requirements. Postoperative angiographic outcomes were evaluated using the modified Thrombolysis in Cerebral Infarction (mTICI) grading system, with mTICI ≥2b considered indicative of successful reperfusion (Figures 1A, B). Immediately following the surgical intervention, a head DSA Dyna-CT scan was performed to confirm the absence or presence of postoperative hemorrhage or contrast extravasation. Following successful recanalization, intravenous nicardipine was administered when adequate blood pressure control could not be achieved. Serial head CT scans were performed to monitor for postoperative complications. Antithrombotic therapy was administered according to individualized clinical indications, initiated only in patients with clear indications (e.g., cardioembolic stroke), based on neurological status and follow-up imaging findings. For patients diagnosed with MBE, prompt communication with family members was undertaken to discuss the potential need for decompressive craniectomy or hematoma evacuation.

### Contrast extravasation

Contrast agent extravasation primarily results from disruption of the local BBB and impaired venous drainage, which together lead to delayed clearance of contrast material from vascular and extravascular compartments under normal hemodynamic conditions. Consequently, hyperdense areas may persist on post-procedural imaging. Characteristic imaging features include high-density opacities distributed along the subarachnoid spaces within the infarcted territory or over the cortical surface and are typically not associated with significant mass effect or surrounding vasogenic edema (19, 22). Unlike other causes of hyperdensity, the key feature of contrast extravasation is that it reflects iodine deposition rather than blood accumulation, and its attenuation generally decreases on short-term follow-up imaging. In contrast, luxury perfusion represents a hemodynamic phenomenon rather than structural leakage. It is typically characterized by thin gyriform or cortical ribbon–like enhancement along the infarcted cortex. The degree of hyperdensity is usually mild, does not form a discrete parenchymal deposit, and does not indicate retention of contrast material within the tissue. Hyperdensity due to severe infarction usually reflects extensive BBB disruption within the deep gray matter, allowing contrast material to enter the infarct core. These hyperdense areas are confined to the vascular territory involved. Although they may appear relatively dense, they generally do not produce significant mass effect or structural displacement unless true hemorrhagic transformation occurs, and their attenuation similarly decreases on follow-up imaging in the absence of hemorrhage. By comparison, subarachnoid hemorrhage typically presents as persistent hyperdensity within the cortical sulci or basal cisterns, often demonstrating a pattern consistent with blood layering and potentially accompanied by secondary complications such as hydrocephalus. In contrast to contrast extravasation, the attenuation of subarachnoid hemorrhage does not rapidly decline over a short interval.

Following MT, residual contrast extravasation is frequently visualized on immediate Dyna-CT scans (Figure 1C). In some cases, hemorrhage may coexist with contrast leakage, making differentiation challenging. When visual assessment is inconclusive, quantitative Hounsfield unit (HU) measurement can assist in distinguishing the two entities: an attenuation value exceeding 100 HU in the affected area strongly suggests contrast extravasation, whereas hemorrhage typically demonstrates values between 60 and 80 HU. If diagnostic uncertainty persists, DECT performed 6–12 h after the procedure can reliably differentiate hemorrhage from contrast extravasation (Figure 1D) (20).

### Statistical analysis

All statistical analyses were performed using SPSS software (version 26.0; IBM Corp., Armonk, NY, USA). Continuous variables were expressed as mean ± SD for normally distributed data or as median (IQR) for non-normally distributed data. Differences between groups were assessed using the independent-samples *t*-test for normally distributed variables and the Mann–Whitney *U* test for non-normally distributed variables. Categorical variables were presented as counts and percentages, and intergroup comparisons were performed using the chi-square (χ^2^) test. Variables with a *P* value < 0.05 in univariate analysis were subsequently entered into multivariate logistic regression to identify independent predictors of postoperative MBE and to construct a predictive model. Model performance was evaluated using receiver operating characteristic (ROC) curve analysis, with the area under the curve (AUC) representing the model's discriminative ability. Model calibration was assessed by the Hosmer–Lemeshow goodness-of-fit test. *P* value < 0.05 was considered statistically significant.

## Results

### Demography data of patients

The final study cohort consisted of 174 patients who underwent endovascular MT for AC-LVO within 24 h of onset. Most patients had a baseline ASPECTS ≥ 6; however, six patients with ASPECTS scores of 3–5 were also included because their infarct core volume was < 50 ml. Among them, 99 patients (56.9%) were male, with a median age of 71 years. Treatment strategies included aspiration-only thrombectomy in 27 cases and stent retriever–based thrombectomy combined with aspiration in 147 cases. Dyna-CT imaging was performed within 30 min of successful recanalization, followed by DECT scans 6–12 h postoperatively. Postoperative MBE occurred in 41 patients (23.6%). As summarized in Table 1, patients in the MBE group demonstrated significantly larger ICV and IPV, higher NIHSS scores, and a greater number of thrombectomy passes compared with the non-MBE group. In contrast, favorable preoperative collateral circulation was associated with a protective effect against the development of postoperative MBE. Moreover, there was a significant difference in 90-day mRS scores between the two groups (*P* < 0.05).

**Table 1:** Comparison of the characteristics of patients with and without MBE.

**Variables**	**Total (*n* = 174)**	**Non-MBE** **(*n* = 133)**	**MBE (*n* = 41)**	**Statistic**	***P***
Age	71.00 (63.25, 76.75)	71.00 (64.00, 76.00)	69.00 (62.00, 77.00)	*Z* = −0.68	0.497
Male	99 (56.90)	72 (54.14)	27 (65.85)	χ^2^ = 1.75	0.185
Smoking	34 (19.54)	26 (19.55)	8 (19.51)	χ^2^ = 0.00	0.996
Drinking	29 (16.67)	23 (17.29)	6 (14.63)	χ^2^ = 0.16	0.69
Diabetes	40 (22.99)	32 (24.06)	8 (19.51)	χ^2^ = 0.37	0.545
Hypertension	118 (67.82)	91 (68.42)	27 (65.85)	χ^2^ = 0.09	0.758
Dyslipidemia	19 (10.92)	16 (12.03)	3 (7.32)	χ^2^ = 0.31	0.576
Coronary artery disease	16 (9.20)	12 (9.02)	4 (9.76)	χ^2^ = 0.00	1.000
Atrial fibrillation	90 (51.72)	68 (51.13)	22 (53.66)	χ^2^ = 0.08	0.777
Stroken	21 (12.07)	19 (14.29)	2 (4.88)	χ^2^ = 1.80	0.179
ICV	47.80 (34.50, 70.38)	41.90 (34.10, 62.30)	68.40 (56.04, 93.70)	*Z* = −4.79	**<0.001**
IPV	176.40 (113.85, 249.55)	149.50 (101.80, 234.00)	221.00 (176.60, 322.00)	*Z* = −3.88	**<0.001**
NIHSS	12.00 (8.00, 16.00)	12.00 (7.00, 15.00)	15.00 (10.00, 17.00)	*Z* = −2.92	**0.003**
Thrombectomy passes	2.00 (1.00, 2.00)	2.00 (1.00, 2.00)	2.00 (1.00, 3.00)	*Z* = −3.03	**0.002**
Contrast extravasation	58 (33.33)	32 (24.06)	26 (63.41)	χ^2^ = 21.84	**<0.001**
Collateral circulation	127 (72.99)	109 (81.95)	18 (43.90)	χ^2^ = 23.02	**<0.001**
Intravenous thrombolysis	50 (28.74)	38 (28.57)	12 (29.27)	χ^2^ = 0.01	0.931
90 d mRS	2.44 ± 1.68	1.90 ± 1.45	4.20 ± 1.05	*t* = −11.07	**<0.001**

Values in bold indicate *P* < 0.05. MBE, malignant brain edema; ICV, infarct core volume; IPV, ischemic penumbra volume; mRS, modified rankin scale; *Z*, Mann–Whitney test; χ^2^, Chi-square test; *t, t*-test.

### Logistic regression analysis

Multivariable logistic regression analysis was performed, incorporating ICV, IPV, number of thrombectomy attempts, baseline NIHSS score, contrast extravasation, and collateral circulation (Table 2). The results demonstrated that ICV (OR = 1.03, 95% CI: 1.01–1.04, *P* < 0.001), NIHSS score (OR = 1.11, 95% CI: 1.01–1.21, *P* = 0.032), number of thrombectomys passes (OR = 1.61, 95% CI: 1.03–2.54, *P* = 0.038), contrast extravasation (OR = 3.84, 95% CI: 1.45–10.18, *P* = 0.007), and collateral scirculation (OR = 0.23, 95% CI: 0.08–0.61, *P* = 0.003) were independent predictors of postoperative MBE. These findings indicate that larger infarct core volume and the presence of contrast extravasation are significantly associated with the development of MBE following successful recanalization in patients undergoing MT.

**Table 2:** Multivariable logistic regression of MBE.

**Variables**	**β**	**S.E**	**Z**	***P***	**OR (95%CI)**
Male	0.26	0.53	0.49	0.621	1.30 (0.46–3.67)
Age	−0.01	0.02	−0.61	0.540	0.99 (0.94–1.03)
Stroken	−1.82	1.05	−1.74	0.082	0.16 (0.02–1.26)
ICV	0.03	0.01	3.67	**<0.001**	1.03 (1.01–1.04)
IPV	0.00	0.00	1.13	0.257	1.00 (1.00–1.01)
NIHSS	0.10	0.05	2.14	**0.032**	1.11 (1.01–1.21)
Thrombectomy passes	0.48	0.23	2.08	**0.038**	1.61 (1.03–2.54)
Contrast extravasation	1.35	0.50	2.71	**0.007**	3.84 (1.45–10.18)
collateral circulation	−1.49	0.51	−2.93	**0.003**	0.23 (0.08–0.61)

Values in bold indicate *P* < 0.05. MBE, malignant brain edema; ICV, infarct core volume; IPV, ischemic penumbra volume; OR, odds ratio; CI, confidence interval.

### Prediction model

**Figure 2:**
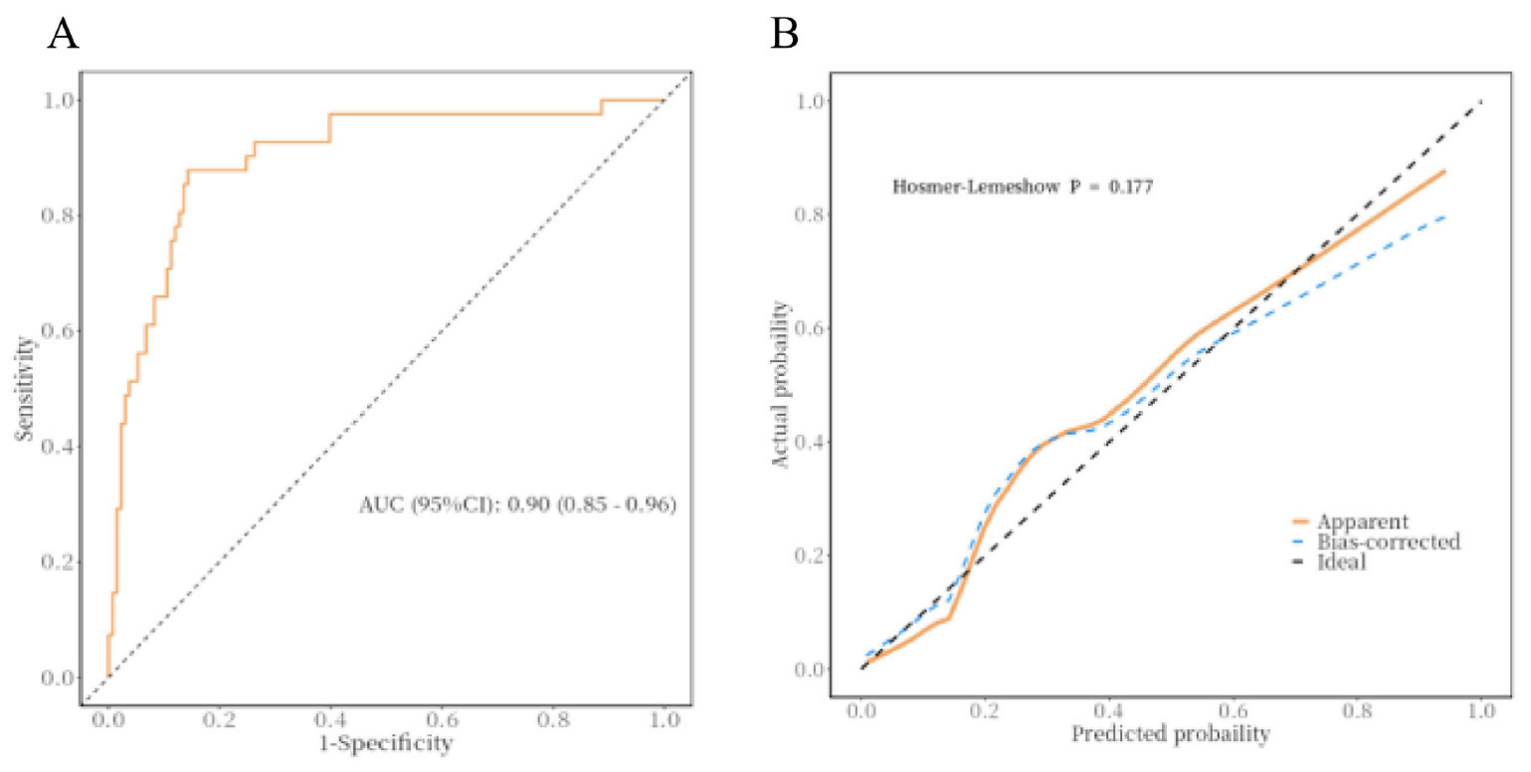
**(A, B)** ROC and calibration curves of the predictive model. ROC, receiver operating characteristic.

After adjusting for age and sex, a multivariable logistic regression model was constructed using ICV, number of thrombectomy passes, baseline NIHSS score, contrast extravasation, and collateral circulation as independent predictors. The model demonstrated excellent discriminative performance (AUC = 0.90, 95% CI: 0.85–0.96) and good calibration according to the Hosmer–Lemeshow goodness-of-fit test (χ^2^ = 11.45, *P* = 0.177, Figure 2).

## Discussion

This study utilized immediate post-MT Dyna-CT imaging data to demonstrate that contrast extravasation, identifiable through distinct visual imaging features, represents a direct and independent risk factor for the development of MBE. Taking MBE as the primary outcome, the study systematically evaluated the predictive value of contrast agent extravasation in stroke patients following MT and established a robust predictive model.

Song et al. (22) employed non-enhanced CT imaging and quantified the ratio of the contrast extravasation area to the ipsilateral cerebral hemisphere volume after MT, proposing that a ratio exceeding 0.2 was associated with a higher probability of developing MBE within 6–22 h. These findings suggest that the extent of contrast leakage may serve as an early imaging marker for MBE and unfavorable clinical outcomes—a result that aligns with the methodology and conclusions of the present study. In conventional CT imaging, differentiation between intracranial hemorrhage and contrast agent retention following MT remains challenging due to their similar hyperdense appearance. Notably, contrast extravasation often conforms to the anatomical distribution of the subarachnoid space, sulci, and gyri, reflecting the high permeability and rapid extravasation kinetics of contrast media compared with blood (23, 24). Ogata et al. (14) further confirmed that the distribution characteristics of contrast extravasation following MT are consistent with localized exudation, typically without a significant mass effect or evident perilesional edema. Contrast extravasation demonstrates regional specificity, with hyperdense iodine distribution predominantly confined to the vascular territory supplied by the previously occluded artery, particularly in areas where the catheter was positioned and contrast was delivered during the procedure. In the present study, although Dyna-CT provides lower image quality compared with DECT, its integration within the angiography suite enables seamless imaging immediately after thrombectomy without additional patient transfer. This confers significant advantages in real-time assessment, procedural simplicity, and bedside accessibility, thereby improving overall workflow efficiency. Nevertheless, the characteristic imaging features of contrast extravasation on Dyna-CT, as well as the underlying pathophysiological mechanisms, warrant further investigation to enhance the diagnostic sensitivity and specificity of this imaging marker in clinical practice.

The primary pathophysiological driver of malignant brain edema is extensive and sustained cerebral ischemia, which leads to energy failure, ionic pump dysfunction, and cytotoxic edema within the infarct core (25). Early disruption of the BBB plays a pivotal role in the development of MBE following MT in acute ischemic stroke (26, 27). Preprocedural factors such as a larger ICV, poor collateral circulation, and prolonged ischemic duration may predispose patients to progressive BBB disruption. Furthermore, repeated mechanical irritation of the vascular endothelium during MT, combined with hyperperfusion injury following reperfusion, can exacerbate endothelial tight junction breakdown, trigger neutrophil activation, and promote the release of matrix metalloproteinases, thereby markedly increasing BBB permeability (15). In this context, contrast extravasation should be interpreted as a downstream imaging manifestation of severe ischemic damage and BBB decompensation, rather than an independent initiating event. Iodinated contrast media may leak from compromised vessels into the brain parenchyma and persist there, manifesting as visible high-density lesions on imaging. This phenomenon not only reflects the onset of vasogenic edema but also signifies that BBB disruption has exceeded compensatory capacity, facilitating fluid infiltration into the interstitial space and leading to elevated intracranial pressure (28). Moreover, contrast agents themselves may exacerbate cerebral injury through osmotic stress and direct cytotoxicity. Toshiharu (29) reported that the hypertonicity and chemical toxicity of iodinated contrast media could induce distal cerebral artery vasospasm and regional hypoperfusion, resulting in prolonged contrast retention within the interstitial compartment and delayed clearance. Collectively, these pathophysiological processes contribute to the development of post-MT MBE. Therefore, contrast extravasation following MT should not be regarded merely as a passive indicator of BBB injury but rather as an active participant in the cascade leading to MBE.

Contrast extravasation not only reflects the hyperperfusion state induced by vascular recanalization but also serves as an indirect imaging marker of cerebral autoregulation (CA) dysfunction. Impaired CA weakens the brain's ability to regulate cerebral blood flow in response to blood pressure fluctuations, resulting in capillary hypertension, microcirculatory dysregulation, and consequently increased BBB permeability and cerebral edema (30). Although MT restores macrovascular perfusion, CA often remains markedly impaired and is closely associated with neurological deterioration, hemorrhagic transformation, the need for decompressive surgery, and poor outcomes (31). Meanwhile, the hypertonicity and cytotoxicity of iodinated contrast agents may amplify this pathological cascade. In patients undergoing MT within the late time window, early formation of microthrombi in the venous microcirculation may persist even after successful arterial recanalization, causing venous drainage dysfunction and consequently increasing the risk of hemorrhagic transformation and MBE (18). Conversely, robust collateral circulation mitigates these changes by preserving CA function, reducing inflammatory responses, and preventing microvenous thrombosis (32). Collectively, these findings suggest that contrast extravasation represents not only a static imaging manifestation of BBB disruption, but also a potential indicator of microcirculatory imbalance secondary to impaired CA. This phenomenon is closely linked to hemorrhagic transformation and MBE, and may serve as a novel imaging biomarker for evaluating reperfusion quality and microvascular integrity in patients after MT.

This study identified ICV, baseline NIHSS score, number of thrombectomy passes, and preoperative collateral circulation as significant predictors of MBE. These findings highlight the multifactorial interplay between preoperative clinical status and intraoperative procedural factors in the pathogenesis of MBE. However, in current clinical practice, the success of MT is often judged solely by the attainment of angiographic recanalization—a conventional paradigm summarized as “recanalization equals success.” Notably, Zhang et al. (7) reported that approximately 11.5% of patients still developed MBE despite successful recanalization, with poor collateral circulation and multiple thrombectomy passes identified as independent risk factors. These results underscore the necessity of shifting from a unidimensional evaluation of recanalization toward a comprehensive, multidimensional framework for risk stratification and outcome prediction following MT. Furthermore, the prediction model developed based on these risk factors demonstrated excellent performance (AUC = 0.90, 95% CI: 0.85–0.96), which indicates a high discriminative ability for identifying patients at elevated risk of MBE. Notably, unlike previous models that primarily relied on clinical scores or volumetric imaging parameters, the inclusion of postoperative contrast extravasation as an imaging variable provides valuable insights into BBB integrity and microcirculatory function following reperfusion. This integration enhances the model's ability to capture the pathophysiological continuum of MBE development after MT. For patients identified as being at high risk of malignant brain edema who demonstrate contrast extravasation on post-MT Dyna-CT, intensified neurological monitoring and serial imaging follow-up are recommended to allow timely detection of edema progression or neurological deterioration. In parallel, hemodynamic management should be optimized, avoiding excessive hypertension that may exacerbate reperfusion injury while also preventing hypotension that could worsen ischemic damage. Early decompressive treatment may be cautiously considered on the basis of ongoing clinical assessment and dynamic imaging evaluation.

However, this study has several limitations. First, as a single-center retrospective analysis, it may be susceptible to selection bias. Second, the quantitative evaluation of contrast extravasation has not yet been standardized, which may affect the reproducibility and reliability of the findings. Third, although imaging biomarkers were the primary focus of this study, clinical assessment variables were not incorporated into the predictive analysis, which may limit the integration of imaging findings with real-world clinical decision-making. Therefore, future multicenter prospective studies are warranted to validate these results and to identify more precise and robust predictive biomarkers for MBE following MT.

## Conclusion

This study demonstrates that contrast extravasation is an independent predictor of MBE following MT for AC-LVO. The predictive model, which integrates contrast extravasation, baseline NIHSS score, ICV, number of thrombectomy passes, and collateral circulation status, exhibited strong predictive performance. As an easily obtainable imaging biomarker, contrast extravasation can be rapidly assessed on-table, avoiding patient transfer, with minimal procedural burden and a low complication profile. It enables early identification of high-risk patients without interrupting clinical workflow, thereby supporting the timely implementation of preventive and individualized management strategies in clinical practice.

## Data Availability

The original contributions presented in the study are included in the article/supplementary material, further inquiries can be directed to the corresponding authors.
